# Altered neural intrinsic oscillations in patients with multiple sclerosis: effects of cortical thickness

**DOI:** 10.3389/fneur.2023.1143646

**Published:** 2023-09-25

**Authors:** Xiao Liang, Lei Wang, Yanyan Zhu, Yao Wang, Ting He, Lin Wu, Muhua Huang, Fuqing Zhou

**Affiliations:** ^1^Department of Radiology, The First Affiliated Hospital of Nanchang University, Nanchang, Jiangxi, China; ^2^Institute of Medical Imaging, Nanchang University, Nanchang, Jiangxi, China

**Keywords:** multiple sclerosis, fMRI, two-dimensional fALFF, cortical thickness, machine learning

## Abstract

**Objective:**

To investigate the effects of cortical thickness on the identification accuracy of fractional amplitude of low-frequency fluctuation (fALFF) in patients with multiple sclerosis (MS).

**Methods:**

Resting-state functional magnetic resonance imaging data were collected from 31 remitting MS, 20 acute MS, and 42 healthy controls (HCs). After preprocessing, we first calculated two-dimensional fALFF (2d-fALFF) maps using the DPABISurf toolkit, and 2d-fALFF per unit thickness was obtained by dividing 2d-fALFF by cortical thickness. Then, between-group comparison, clinical correlation, and classification analyses were performed in 2d-fALFF and 2d-fALFF per unit thickness maps. Finally, we also examined whether the effect of cortical thickness on 2d-fALFF maps was affected by the subfrequency band.

**Results:**

In contrast with 2d-fALFF, more changed regions in 2d-fALFF per unit thickness maps were detected in MS patients, such as increased region of the right inferior frontal cortex and faded regions of the right paracentral lobule, middle cingulate cortex, and right medial temporal cortex. There was a significant positive correlation between the disease duration and the 2d-fALFF values in the left early visual cortex in remitting MS patients (*r* = 0.517, Bonferroni-corrected, *p* = 0.008 × 4 < 0.05). In contrast with 2d-fALFF, we detected a positive correlation between the 2d-fALFF per unit thickness of the right ventral stream visual cortex and the modified Fatigue Impact Scale (MFIS) scores (*r* = 0.555, Bonferroni-corrected, *p* = 0.017 × 4 > 0.05). For detecting MS patients, 2d-fALFF and 2d- fALFF per unit thickness both performed remarkably well in support vector machine (SVM) analysis, especially in the remitting phase (AUC = 86, 83%). Compared with 2d-fALFF, the SVM model of 2d-fALFF per unit thickness had significantly higher classification performance in distinguishing between remitting and acute MS. More changed regions and more clinically relevant 2d-fALFF per unit thickness maps in the subfrequency band were also detected in MS patients.

**Conclusion:**

By dividing the functional value by the cortical thickness, the identification accuracy of fALFF in MS patients was detected to be potentially influenced by cortical thickness. Additionally, 2d-fALFF per unit thickness is a potential diagnostic marker that can be utilized to distinguish between acute and remitting MS patients. Notably, we observed similar variations in the subfrequency band.

## 1. Introduction

Multiple sclerosis (MS) is an immune-mediated chronic inflammatory demyelinating disease of the central nervous system (1), and it is labeled as “highly disabling” and “burdensome” (2). The clinical symptoms of MS are complex and varied, commonly featuring severe cognitive and motor impairments. Functional magnetic resonance imaging (fMRI) technology, which is regarded to hold promise for bridging the “imaging and clinical disparity” in MS patients, has been developed (3). Various fMRI analysis methods, such as graph theory analysis, independent component analysis, regional homogeneity, and functional connectivity, have provided new evidence for the pathophysiological mechanism of MS (4–7). For example, a trend toward possibly beneficial higher fMRI activity at early MS stages and maladaptive decreased fMRI activity later on in the disease is characterized by a progressive collapse of long-range connections and impaired hub integration (8, 9), driving motor and cognitive disability through a loss of network efficiency (10). However, when MS participants were examined, the resting-state fMRI results were only partially consistent. Numerous functional connectivity abnormalities were observed in MS patients; some studies indicated a notable rise in global connectivity levels (11–14), while others reported a decline in functional connectivity (15, 16). Although there are indications that specific connectivity patterns are peculiar to distinct disease stages (17), longitudinal works remain rare. Interestingly, a previous finding suggested that when gray matter volume was employed as covariate, the sex-related increase in functional connectivity faded (18). Therefore, we doubt whether structure changes affect the observation of functional changes in MS patients.

Although functional connectivity analysis can provide us with more holistic information on a set of brain regions within a network, it does not reveal blood oxygenation level-dependent (BOLD) signal changes in regional spontaneous activity. As the amplitude of low-frequency fluctuation (ALFF) is sensitive to physiological noise, the fractional ALFF (fALFF) ratio of the low-frequency power spectrum (0.01–0.1 Hz) to the entire frequency range can be used to effectively suppress the non-specific signal components of resting-state fMRI, greatly increasing the sensitivity and specificity of detecting spontaneous activity in different brain regions (19, 20). Additionally, surface-based two-dimensional fALFF (2d-fALFF) uses tightly linked regional variables for cortical alignment, such as folding patterns, enhancing regional alignment and increasing specificity and retest reliability (21). Importantly, 2d-fALFF can serve as a powerful biomarker that reflects neuropathological changes in the disease (22). Moreover, Sailer and colleagues found a significant overall thinning of the cerebral cortex in MS patients compared with healthy controls (23).

In this study, we hypothesized that the identification accuracy of fALFF in MS patients may be potentially influenced by cortical thickness. This effect may significantly affect the results of between-group comparisons, clinical correlations, and classification analyses of MS patients. To test these hypotheses, first, as most previous MS research has concentrated on the damage that occurs during the remitting phase, it is unclear whether the neuronal oscillatory activity in the brain differs between the acute phase and remitting phase of MS. Therefore, we split MS patients into acute and remitting phases. Second, between-group comparison, clinical correlation, and classification analyses were performed in 2d-fALFF and 2d-fALFF per unit thickness maps.

Furthermore, the human brain’s neural oscillations in various frequency bands may be responsive to the activity of various brain areas and serve as a useful indicator of various physiological functions of brain activity (24–27). The frequency spectrum was decomposed into five different frequency bands: slow-6 (0–0.01 Hz), slow-5 (0.01–0.027 Hz), slow-4 (0.027–0.073 Hz), slow-3 (0.073–0.198 Hz), and slow-2 (0.198–0.25 Hz). The slow-6, slow-3, and slow-2 oscillations mostly reflect very low-frequency drift, white matter signals, and high-frequency physiological noise, respectively. To find links between functional processing and illnesses, slow-4 and slow-5 oscillations are useful and largely connected with gray matter (25, 28). Therefore, we also calculated the between-group comparison, clinical correlation, and classification analysis of MS patients in the subfrequency band. Studying the neural oscillations performed in 2d-fALFF and 2d-fALFF per unit thickness maps could offer new insights into fMRI studies of MS.

## 2. Materials and methods

### 2.1. Participants

A total of 51 MS patients and 42 healthy controls (HCs) with approximately matched sex and age were recruited from the First Affiliated Hospital of Nanchang University and the local community from 2011 to 2021. The inclusion criteria for patients were as follows: (i) age 20–65 years and (ii) clinically confirmed and reviewed by the revised diagnostic criteria of McDonald in 2017 (29). The exclusion criteria for patients were as follows: (i) a history of head injury or other neuropsychiatric diseases; (ii) other autoimmune diseases; (iii) contraindications to MRI scanning; (iv) poor image quality; (v) excessive head motion >2.5 mm and rotation >2.5°; and (vi) incomplete clinical-scale assessment information. The inclusion criteria for HCs were as follows: (i) age 20–65 years; (ii) no previous or current mental illness and definite physical illness; (iii) no family history of mental illness. The HC exclusion criteria for HCs were as follows: (i) contraindications to MRI scanning; (ii) poor image quality; (iii) excessive head motion >2.5 mm and rotation >2.5°.

Subsequently, we divided the MS patient group into the acute phase and remitting phase. The acute phase mainly met several criteria: active attack or functional impairment lasting at least 24 h and/or gadolinium-enhancing lesion. The remitting phase met the following criterion: had not experienced a relapse in the 3 months preceding the MRI measurement.

### 2.2. Clinical and neuropsychological assessments

All MS patients were evaluated by the following scales: (i) Paced Auditory Serial Addition Test (PASAT) to detect the degree of impaired cognitive information processing speed; (ii) Expanded Disability Status Scale (EDSS) to assess the cumulative disability status of the current disease, where higher scores indicate a more serious degree of neurological deficit (EDSS score ≤ 2.5 is low disability, between 3 and 6 is moderate disability, and EDSS score ≥ 6.5 is severe disability); (iii) Modified Fatigue Impact Scale (MFIS) scale, which mainly measures the effects of fatigue on the cognitive, physical, and psychosocial functions of MS patients.

### 2.3. MR imaging acquisition

MRI data were acquired using a Trio 3.0 T MRI scanner and an 8-channel phase-controlled head coil (Siemens, Munich, Germany). During the scan, each subject was kept in a supine position with foam pads used to reduce head movement, wore earplugs, and was asked to close their eyes, relax, and remain awake with minimal specific thinking. Resting-state fMRI (rs-fMRI) images, high-resolution 3D T1-weighted and CE T1-weighted images were obtained for each participant using the following sequences:

- rs-fMRI image acquisition was performed using an echo planar imaging sequence with gradient echo: repetition time (TR)/echo time (TE) = 2000/30 ms, matrix = 64 × 64, field of view (FOV) = 210 × 210 mm, slice thickness = 4.00 mm, and 240 time points.
- High-resolution 3D T1-weighted image acquisition was performed with TR/TE = 1900/2.26 ms, matrix = 240 × 256, FOV = 215 × 230 mm, slice thickness = 1.00 mm, and 176 sagittal slices. After scanning, the scanning was enhanced by 0.1 mmol/kg gadopentetate dimeglumine (Gd-DTPA).

### 2.4. Data preprocessing

The following preprocessing was performed on the MATLAB R2018b (Math Works, Inc.) platform using the DPABISurf_V1.6 toolbox^1^ for the structural and functional data of each subject (30).

The processing steps for structural images included (i) spatially adaptive non-local mean filtering used to remove MRI space noise (31); (ii) intensity correction; (iii) skull stripping; (iv) brain tissue segmentation: cerebrospinal fluid (CSF), white matter (WM), and gray matter (GM); (v) brain surface reconstruction by the “recon-all” command in FreeSurfer 6.0.1 (32); and (vi) spatial normalization in “fsaverage5” space (FreeSurfer reconstruction nomenclature). Based on cortical atlas labels, DPABISurf provided the cortical thickness maps.

The functional image processing steps included (i) removal of the first 10 time points; (ii) skull stripping; (iii) slice-timing correction; (iv) head motion correction; (v) nuisance correction by regressing out the WM and CSF mean time series according to the WM and CSF masks segmented by FreeSurfer and the Friston-24 motion time series, except for the global signal; (vi) a boundary-based registration (BBR) algorithm used to register individual structural and functional images (33); (vii) projection of the individual preprocessed volume-based function image onto a standard cortical surface (fsaverage5); and (viii) finally, smoothing with a 6-mm full-width at half-maximum Gaussian kernel. We determined the midpoints of each pair of vertices on the FREESURFER-created pia meningeal and white matter surfaces. We were able to obtain the registration matrix between each subject’s native fMRI volume and structural volume using the BBR technique. We then selected fsaverage5 surface space as the projection and interpolation target surface. The corresponding coordinates in the native structure space and the corresponding coordinates in the fMRI space for each subject’s vertex in the fsaverage5 space were determined first. The trilinear interpolation method was then used to interpolate the fMRI data based on these provided coordinates.

### 2.5. 2d-fALFF calculation

The 2d-fALFF values were calculated using the DPABISurf_V1.6 toolbox and were obtained as the ratio of each frequency in the low-frequency range to the power in the entire frequency range (0–0.25 Hz). For standardization purposes, the vertex-wise fALFF data for each participant were converted into a *z* score by subtracting the global mean value and then dividing by the standard deviation (SD) of the whole-brain 2d-fALFF. Subsequently, the 2d-fALFF maps were divided by the cortical thickness maps to obtain the 2d-fALFF per unit thickness maps.

In addition to the typical band of 0.01–0.1 Hz, we calculated the 2d-fALFF maps and 2d-fALFF per unit thickness maps in the slow-2 band (0.198–0.25 Hz), slow-3 band (0.073–0.198 Hz), slow-4 band (0.027–0.073 Hz), slow-5 band (0.01–0.027 Hz), and slow-6 band (0–0.01 Hz).

### 2.6. Statistical analysis

To determine the differences between paired subgroups (remitting versus acute MS, acute MS versus HC groups, and remitting MS versus HC groups), the 2d-fALFF values and 2d-fALFF per unit thickness values were analyzed by analysis of variance (ANOVA) using spm12-based DPABISurf statistical analysis with a Tukey–Kramer *post hoc* test (age and sex as covariates, family-wise error (FWE) corrected, vertex *p-*value = 0.001, two-tailed, cluster *p*-value = 0.025).

To examine the clinical correlation, we conducted a partial correlation analysis (age and sex as covariates) between the 2d-fALFF and 2d-fALFF per unit thickness values with the PASAT scale scores, EDSS scale scores, MFIS scale scores, and disease duration in MS patients (P × n < 0.05, Bonferroni correction).

### 2.7. SVM classification analysis

The 2d-fALFF and 2d-fALFF per unit thickness value of all different brain regions between each subgroup in the typical band and subfrequency band were used as ROIs, and an SVM model was constructed to predict the classification of the three groups (remitting versus acute MS, acute MS versus HC groups, and remitting MS versus HC groups). To improve the interpretability of the model, linear kernel functions were considered in this study. The optimal hyperplane was found using the regularization parameter C in the linear kernel support vector machine model. The hyperparameter C retains the default settings and is verified by a 5-fold crossover, with a parameter search range of 2^−10^ to 2^10^ and a step size of 2^0.2^. The optimal model parameters were determined by linear kernel function and hyperparameter search, and the optimal SVM model was selected by cross-validation using the leave-one-out method. Model accuracy, sensitivity, and specificity were calculated, and the performance of the model was evaluated using the receiver operating characteristic (ROC) curve and area under the curve (AUC). The linear SVM model was constructed in MATLAB R2014b using libsvm v3.24.^2^ To investigate the significance of SVM model performance in different frequency bands, the differences between various AUCs of the typical, slow-4, and slow-5 bands were compared using the Delong test (34).

## 3. Results

### 3.1. Demographic and clinical data profiling

A total of 4 remitting MS patients were excluded due to incomplete clinical-scale assessment information. Moreover, 27 remitting MS patients, 20 acute MS patients, and 42 HCs were entered into the final analysis. The demographic and clinical information are shown in Table 1. There were no significant differences between the three groups in terms of age or sex. The median EDSS scores of patients in the remitting and acute MS groups were 1 and 2.5, respectively.

**Table 1:** Demographic and clinical characteristics of HCs and MS patients.

	MS patients		HCs	*P-*value
	Remitting phase (*n* = 27)	Acute phase (*n* = 20)	(*n* = 42)	
Age, years (mean ± SD)	39 ± 9	45 ± 10	44 ± 11	0.590^a^
Sex (M/F)	9/18	7/13	15/27	0.980^b^
EDSS (median)	1.00	2.50	–	<0.001^c^
PASAT (mean ± SD)	90.52 ± 15.23	80.70 ± 18.90	–	0.055^d^
MFIS (mean ± SD)	8.19 ± 3.64	11.15 ± 3.25	–	0.006^d^
Disease duration, months (median)	22.00	13.50	–	0.620^c^

EDSS, expanded disability status scale; PASAT, paced auditory serial addition test; MFIS, modified fatigue impact scale; F, female; M, male; −, no data; SD, standard deviation; ^a^variance test; ^b^chi-square test; ^c^Mann–Whitney *U* test; ^d^two-sample *t-*test.

### 3.2. Between-group comparison

**Figure 1:**
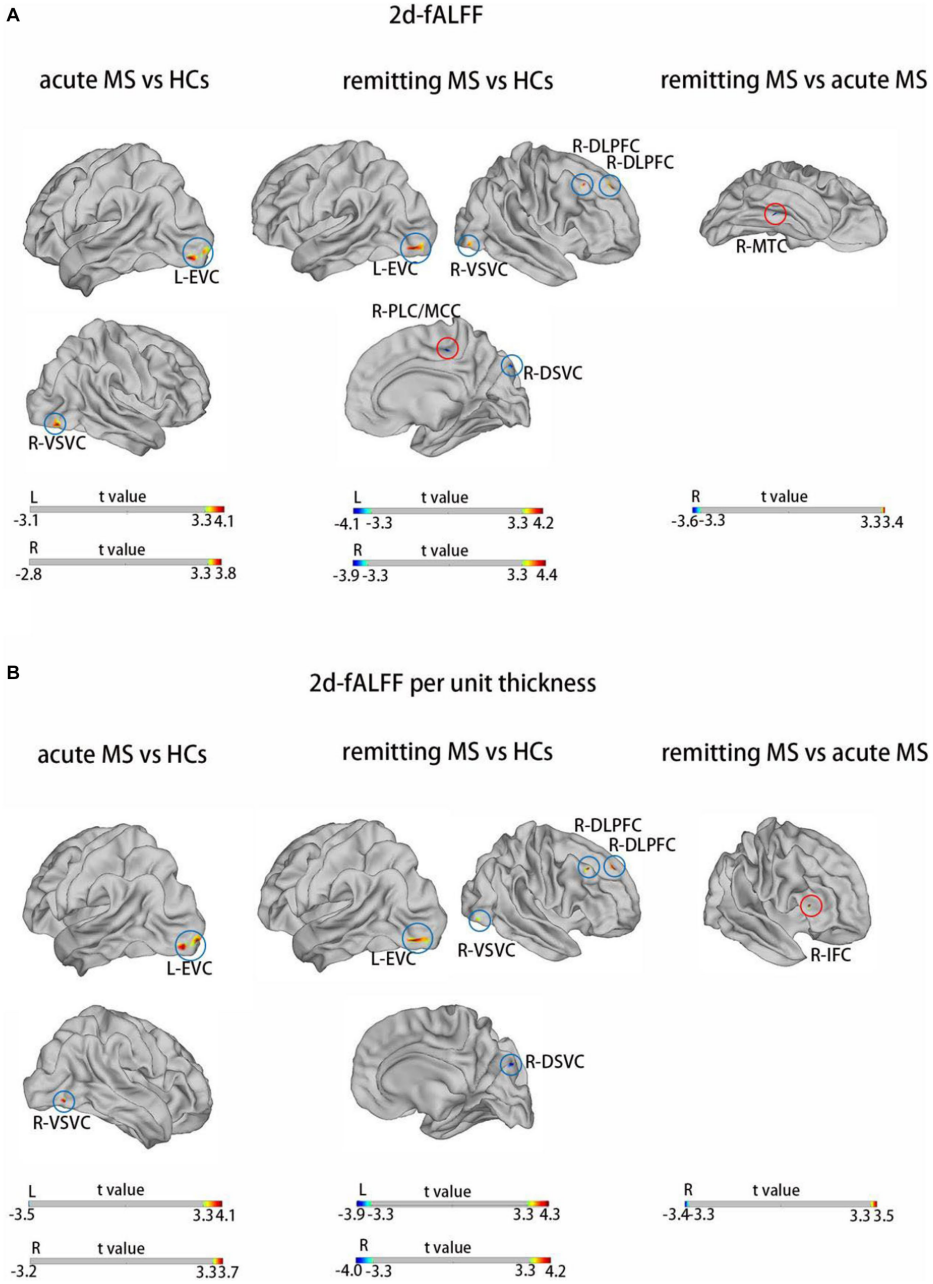
Brain regions with significant differences in 2d-fALFF maps **(A)** and 2d-fALFF per unit thickness maps **(B)** in the typical band among acute MS, remitting MS, and HCs. L, left; R, right; MTC, medial temporal cortex; IFC, inferior frontal cortex; EVC, early visual cortex; VSVC, ventral stream visual cortex; DLPFC, dorsolateral prefrontal cortex; DSVC, dorsal stream visual cortex; PVC, primary visual cortex; red circle: different regions between 2d-fALFF maps and 2d-fALFF per unit thickness maps; blue circle: similar regions between 2d-fALFF maps and 2d-fALFF per unit thickness maps.

Figure 1 and Table 2 show the alteration of the 2d-fALFF of the typical frequency band based on the voxel-based analyses of the acute and remitting MS patients and HCs. In contrast with 2d-fALFF, we can see some changes in 2d-fALFF per unit thickness maps, where fALFF increased from no signal to a positive signal in the right inferior frontal cortex for remitting MS vs. acute MS, where fALFF increased from a negative signal to no signal in the right paracentral lobule and middle cingulate cortex for remitting MS vs. HC, and where fALFF increased from a negative signal to no signal in the right medial temporal cortex in remitting MS vs. acute MS. We also detected between-group differences of brain regions in the cortical thickness maps, as shown in Supplementary Figure S1 and Supplementary Table S1.

**Table 2:** Brain regions with significant differences in 2d-fALFF maps and 2d-fALFF per unit thickness maps in the typical band among acute MS, remitting MS, and HCs.

	Brain regions	HCP	Cluster size (mm^2^)	MNI			Peak intensity
				*X*	*Y*	*Z*	
2d-fALFF in acute MS vs. HCs	L-EVC	5	204.908	−29.45	−93.54	−9.11	4.11
	R- VSVC	22	33.925	44.85	−78.84	−10.92	3.68
2d-fALFF per unit thickness in acute MS vs. HCs	L-PVC	1	209.269	−18.04	−101.63	−4.75	4.11
	R- VSVC	22	33.925	44.85	−78.84	−10.92	3.68
2d-fALFF in remitting MS vs. HCs	L-EVC	6	131.117	−36.44	−89.76	−8.14	4.22
	R-VSVC	22	47.746	44.85	−78.84	−10.92	3.93
	R-DLPFC	67	21.747	36.76	24.28	44.91	4.21
	R-DLPFC	70	56.278	16.77	42.98	40.47	4.37
	R-DSVC	13	22.043	14.62	−87.12	36.81	−3.75
	R-PLC/MCC	37	24.829	12.81	−27.45	47.79	−3.90
2d-fALFF per unit thickness in remitting MS vs. HCs	L-EVC	6	144.758	−36.44	−89.76	−8.14	4.26
	R-VSVC	22	47.746	44.85	−78.84	−10.92	3.71
	R-DLPFC	67	40.540	36.76	24.28	44.91	4.19
	R-DLPFC	70	46.598	16.77	42.98	40.47	4.10
	R-DSVC	13	22.043	14.62	−87.11	36.81	−4.01
2d-fALFF in remitting MS vs. acute MS	R-MTC	135	23.835	44.04	−35.09	−20.66	−3.62
2d-fALFF per unit thickness in remitting MS vs. acute MS	R-IFC	82	21.695	47.77	31.21	8.45	3.53

L, left; R, right; MTC, medial temporal cortex; IFC, inferior frontal cortex; EVC, early visual cortex; VSVC, ventral stream visual cortex; DLPFC, dorsolateral prefrontal cortex; DSVC, dorsal stream visual cortex; PVC, primary visual cortex; HCP, human connectome project; MNI, Montreal Neurological Institute.

**Figure 2:**
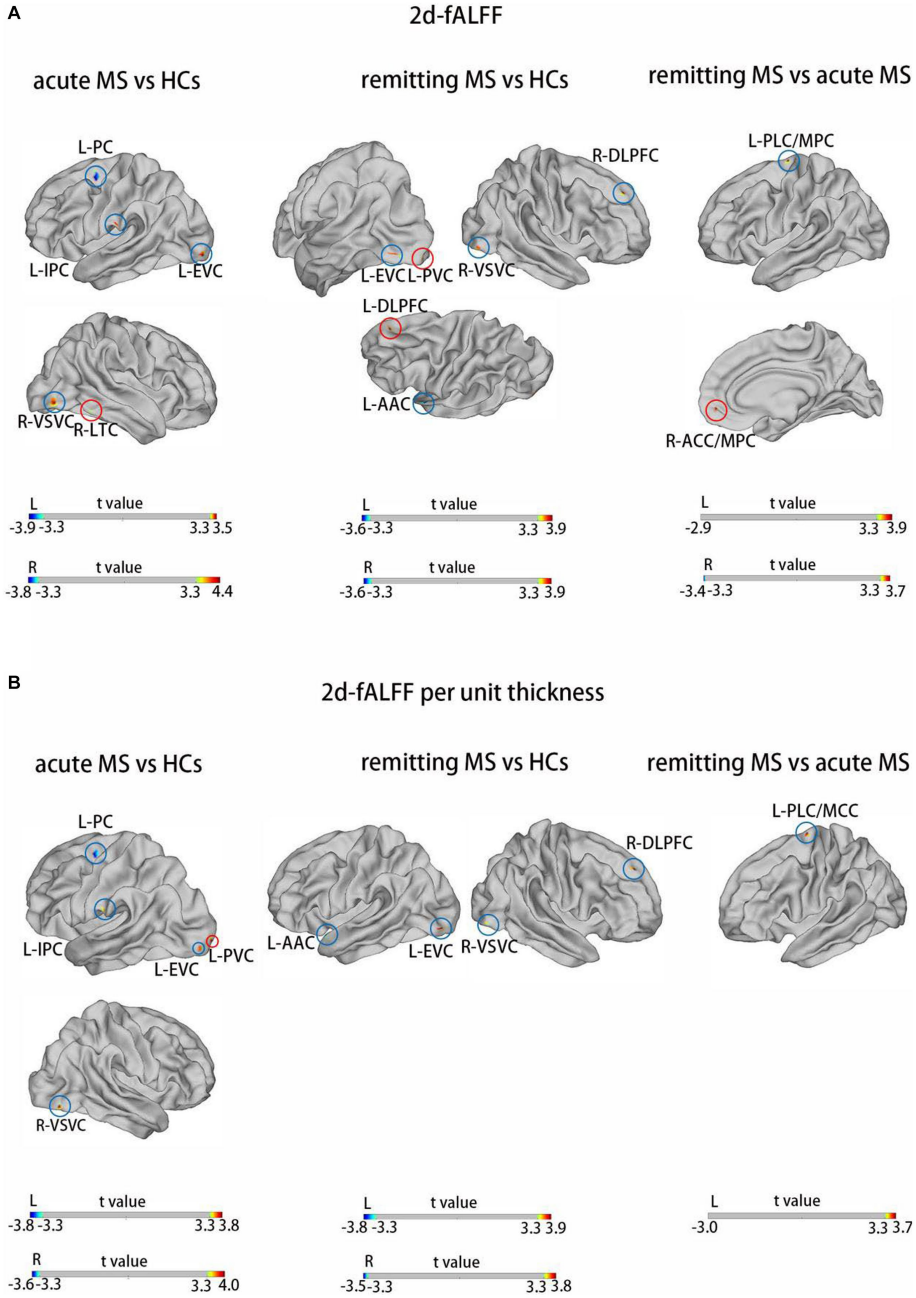
Brain regions with significant differences in 2d-fALFF maps **(A)** and 2d-fALFF per unit thickness maps **(B)** in the slow-4 band among acute MS, remitting MS, and HCs. L, left; R, right; PLC/MCC, paracentral lobular and mid-cingulate cortex; ACC/MPC, anterior cingulate and medial prefrontal cortex; EVC, early visual cortex; AAC, auditory association cortex; DLPFC, dorsolateral prefrontal cortex; VSVC, ventral stream visual cortex; PC, premotor cortex; LTC, lateral temporal cortex; IPC, inferior parietal cortex; red circle: different regions between 2d-fALFF maps and 2d-fALFF per unit thickness maps; blue circle: similar regions between 2d-fALFF maps and 2d-fALFF per unit thickness maps.

**Figure 3:**
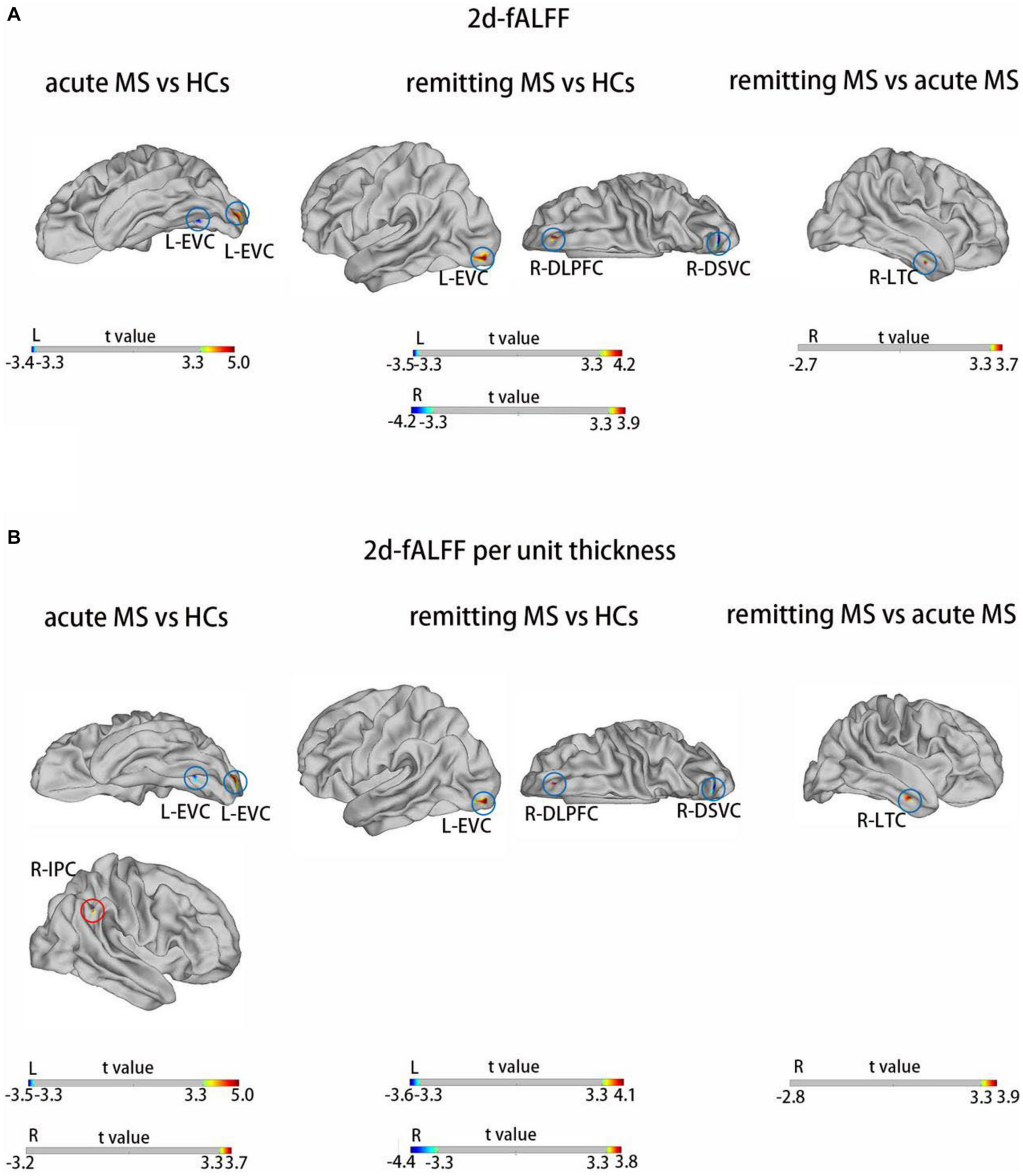
Brain regions with significant differences in 2d-fALFF maps **(A)** and 2d-fALFF per unit thickness maps **(B)** in the slow-5 band among acute MS, remitting MS, and HCs. L, left; R, right; LTC, lateral temporal cortex; EVC, early visual cortex; DSVC, dorsal stream visual cortex; DLPFC, dorsolateral prefrontal cortex; IPC, inferior parietal cortex; red circle: different regions between 2d-fALFF maps and 2d-fALFF per unit thickness maps; blue circle: similar regions between 2d-fALFF maps and 2d-fALFF per unit thickness maps.

Figures 2,3 and Tables 3 4 show the alteration of the 2d-fALFF based on voxel-based and cortical-based analysis in the slow-4 and slow-5 band among acute MS, remitting MS, and HCs. In contrast with 2d-fALFF, we can see some changes in 2d-fALFF per unit thickness maps in slow-4 band, where fALFF increased from no signal to a positive signal in the left primary visual cortex and a positive signal to no signal in the right lateral temporal cortex for acute MS vs. HC, where fALFF increased from a positive signal to no signal in the left primary visual cortex and left dorsolateral prefrontal cortex for remitting MS vs. HC, and where fALFF increased from a positive signal to no signal in the right anterior cingulate and medial prefrontal cortex for remitting MS vs. acute MS. In contrast with 2d-fALFF, we can see some changes in 2d-fALFF per unit thickness maps in slow-5 band, where fALFF increased from a no signal to positive signal in the right inferior parietal cortex for acute MS vs. HC. We also found between-group differences between 2d-fALFF maps and 2d-fALFF per unit thickness maps in the slow-2 band, slow-3 band, and slow-6 band, as shown in Supplementary Figures S2–S4 and Supplementary Tables S2–S4.

**Table 3:** Brain regions with significant differences in 2d-fALFF maps and 2d-fALFF per unit thickness maps in the slow-4 band among acute MS, remitting MS, and HCs.

	Brain regions	HCP	Cluster size (mm^2^)	MNI			Peak intensity
				*X*	*Y*	*Z*	
2d-fALFF in acute MS vs. HCs	L-PC	96	40.755	−20.22	0.47	51.37	−3.92
	L-EVC	4	42.905	−27.28	−95.68	−10.54	3.55
	L-IPC	147	18.796	−57.49	−23.02	25.43	3.50
	R-VSVC	22	96.452	44.84	−80.89	−9.85	4.40
	R-LTC	133	35.219	58.15	−50.00	−18.72	3.45
2d-fALFF per unit thickness in acute MS vs. HCs	L-PC	96	30.399	−20.06	1.76	53.61	−3.80
	L-EVC	4	40.958	−27.28	−95.68	−10.54	3.60
	L-IPC	147	18.796	−58.31	−25.28	24.02	3.53
	L-PVC	1	36.594	−18.04	−101.63	−4.75	3.79
	R-VSVC	22	42.512	44.84	−80.89	−9.85	4.03
2d-fALFF in remitting MS vs. HCs	L-EVC	6	31.690	−36.44	−89.76	−8.14	3.69
	L-AAC	107	31.295	−54.15	6.16	−10.13	−3.62
	L-DLPFC	68	30.440	−14.95	39.74	45.44	3.89
	L-PVC	1	21.971	−17.39	−99.79	−11.71	3.60
	R-DLPFC	70	37.136	16.77	42.98	40.47	3.78
	R-VSV	22	22.283	44.84	−80.89	−9.98	3.41
2d-fALFF per unit thickness in remitting MS vs. HCs	L-EVC	6	43.578	−31.44	−89.76	−8.14	3.87
	L-AAC	107	30.472	−53.58	8.69	−12.10	−3.81
	R-DLPFC	70	37.136	16.77	42.98	40.47	3.78
	R-VSVC	22	22.284	44.84	−80.89	−9.98	3.41
2d-fALFF in remitting MS vs. acute MS	L-PLC/MCC	55	18.754	−11.34	−13.59	−68.32	3.92
	R-ACC/MPC	65	26.662	8.02	49.64	−6.23	3.69
2d-fALFF per unit thickness in remitting MS vs. acute MS	L-PLC/MCC	55	18.754	−11.34	−13.59	−68.32	2.73

L, left hemisphere; R, right hemisphere; PLC/MCC, paracentral lobular and mid-cingulate cortex; ACC/MPC, anterior cingulate and medial prefrontal cortex; EVC, early visual cortex; AAC, auditory association cortex; DLPFC, dorsolateral prefrontal cortex; VSVC, ventral stream visual cortex; PC, premotor cortex; LTC, lateral temporal cortex; IPC, inferior parietal cortex; HCP, human connectome project; MNI, Montreal Neurological Institute.

**Table 4:** Brain regions with significant differences in 2d-fALFF maps and 2d-fALFF per unit thickness maps in the slow-5 band among acute MS, remitting MS, and HCs.

	Brain regions	HCP	Cluster size (mm^2^)	MNI			Peak intensity
				*X*	*Y*	*Z*	
2d-fALFF in acute MS vs. HCs	L-EVC	4	246.459	−27.28	−95.68	−10.54	4.98
	L-EVC	6	46.349	−25.99	−63.97	−6.83	−3.45
2d-fALFF per unit thickness in acute MS vs. HCs	L-EVC	46	254.555	−27.28	−95.68	−10.54	5.01
	L-EVC	6	35.061	−25.99	−63.97	−6.83	−3.48
	R-IPC	149	−26.870	53.23	−46.21	36.35	3.63
2d-fALFF in remitting MS vs. HCs	L-EVC	4	152.421	−27.28	−95.68	−10.54	4.20
	R-DSVC	13	93.254	13.19	−85.90	37.80	−4.15
	R-DLPFC	70	27.409	16.31	40.55	42.31	3.91
2d-fALFF per unit thickness in remitting MS vs. HCs	L-EVC	4	113.297	−27.28	−95.68	−10.54	4.13
	R-DSVC	13	73.736	13.19	−85.90	37.80	−4.44
	R-DLPFC	70	22.198	15.82	38.51	44.92	3.84
2d-fALFF in remitting MS vs. acute MS	R-LTC	134	23.431	47.09	−6.95	−31.63	3.74
2d-fALFF per unit thickness in remitting MS vs. acute MS	R-LTC	134	47.359	47.60	−3.79	−30.23	3.61

L, left hemisphere; R, right hemisphere; LTC, lateral temporal cortex; EVC, early visual cortex; DSVC, dorsal stream visual cortex; DLPFC, dorsolateral prefrontal cortex; IPC, inferior parietal cortex; HCP, human connectome project; MNI, Montreal Neurological Institute.

### 3.3. Correlation analysis

**Figure 4:**
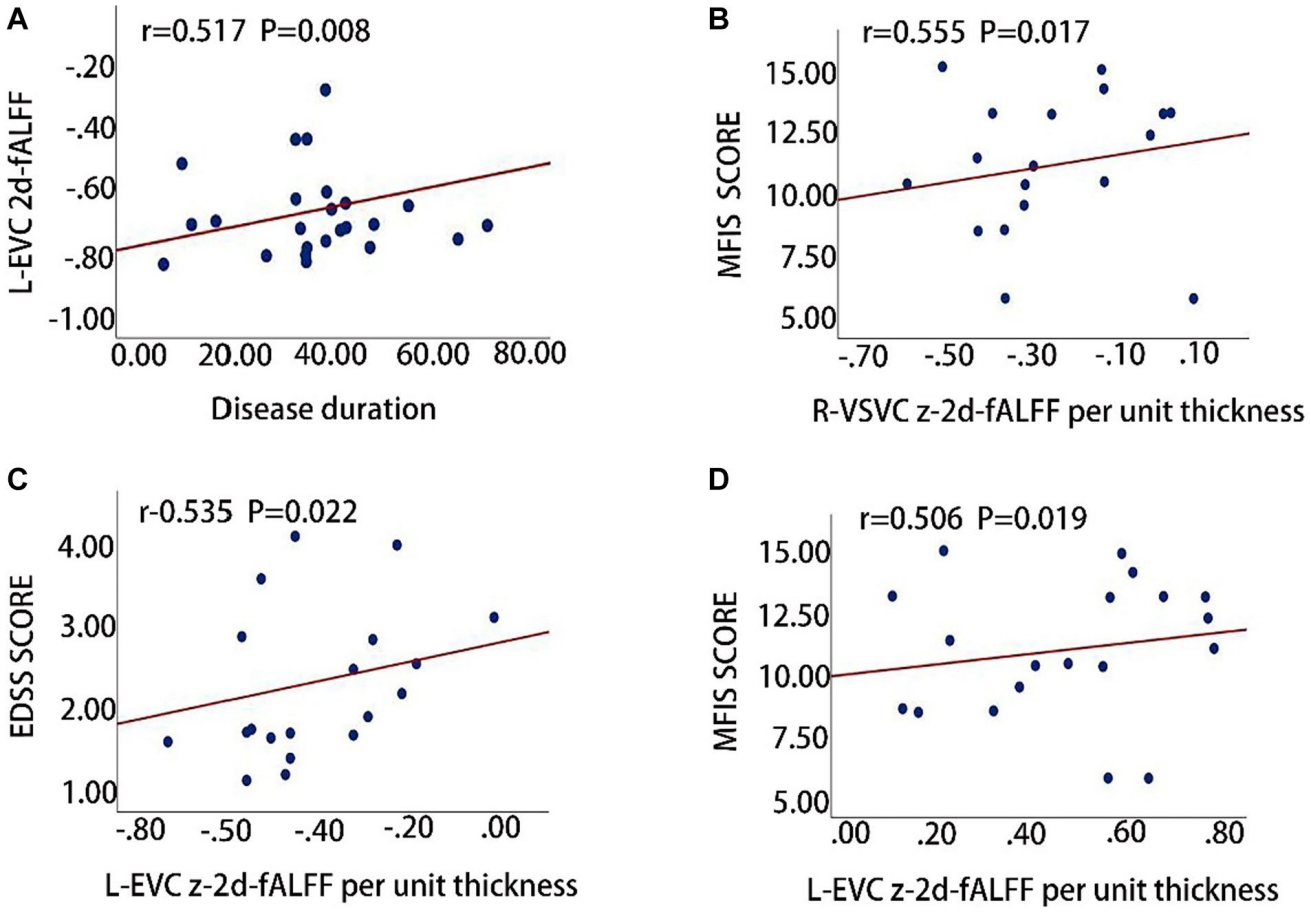
Clinical relevance of 2d-fALFF maps and 2d-fALFF per unit thickness maps in the typical band and slow-5 band among acute MS, remitting MS, and HCs. **(A)** The 2d-fALFF in the left early visual cortex (L-EVC) in the typical band is positively correlated with disease duration in the remitting MS group; **(B)** The 2d-fALFF per unit thickness in the right ventral stream visual cortex (R-VSVC) in the typical band is positively correlated with MFIS scale scores in the acute MS group; **(C,D)** The 2d-fALFF per unit thickness in the L-EVC in the slow-5 band is positively correlated with EDSS scores and MFIS scores.

The clinical relevance of 2d-fALFF maps and 2d-fALFF per unit thickness maps in the typical band among acute MS, remitting MS, and HCs was investigated using partial correlation analysis. There were significantly positive correlations between the disease duration and the 2d-fALFF from the regions of the left early visual cortex in remitting MS patients (*r* = 0.517, Bonferroni-corrected, *p* = 0.008 × 4 < 0.05) (Figure 4A). In contrast with the 2d-fALFF, we detected positive correlations between the 2d-fALFF per unit thickness of the right ventral stream visual cortex and the MFIS scores (*r* = 0.555, Bonferroni-corrected, *p* = 0.017 × 4 > 0.05) (Figure 4B).

We also observed that the 2d-fALFF per unit thickness value of the left early visual cortex in slow-5 band was positively correlated with the EDSS and MFIS scores (*r* = 0.535, Bonferroni-corrected, *p* = 0.022 × 4 > 0.05; *r* = 0.506, Bonferroni-corrected, *p* = 0.019 × 4 > 0.05) (Figures 4C,D).

### 3.4. SVM classification results

**Figure 5:**
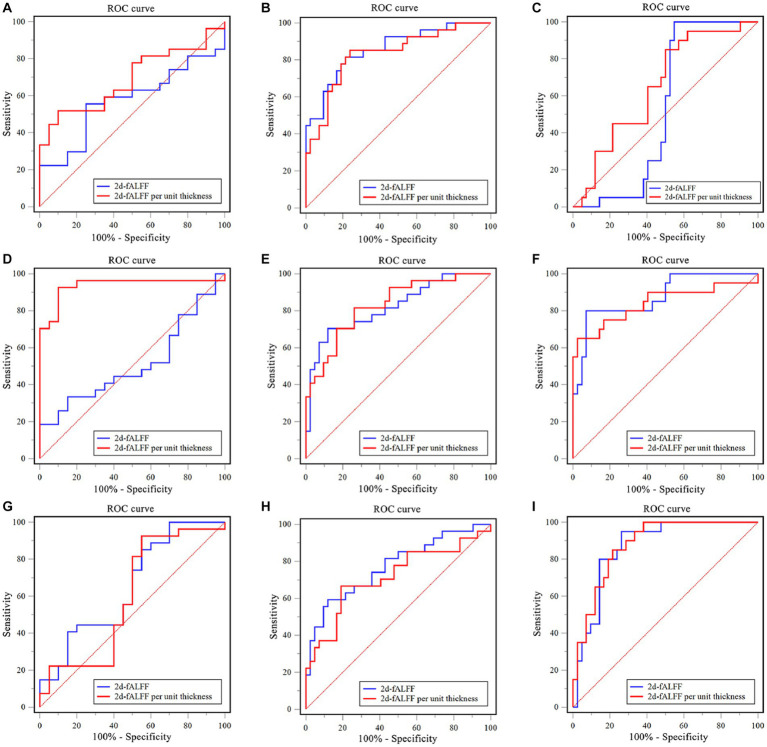
Examination of the receiver operating characteristic (ROC) curve of the classification model between subgroups in the typical band and subfrequency band. **(A)** Remitting multiple sclerosis (MS) vs. acute MS in a typical band; **(B)** Remitting MS vs. healthy controls (HC) in a typical band; **(C)** Acute MS vs. HC in a typical band. **(D)** Remitting MS vs. acute MS in the slow-4 band; **(E)** Remitting MS vs. HC in the slow-4 band; **(F)** Acute MS vs. HC in the slow-4 band. **(G)** Remitting MS vs. acute MS in the slow-5 band; **(H)** Remitting MS vs. HC in the slow-5 band; **(I)** Acute MS vs. HC in the slow-5 band.

The SVM classifier was performed to investigate the classification performance of the 2d-fALFF and 2d-fALFF per unit thickness of the typical frequency band in the acute and remitting MS patients and HCs. Based on the ROI of the values of all brain regions, the SVM model of 2d-fALFF had an area under the curve (AUC) of 0.57, 0.86, and 0.53, as shown in Figures 5A–C. In contrast with 2d-fALFF, the AUC of the SVM model of 2d-fALFF per unit thickness maps was 0.69, 0.83, and 0.65 (Figures 5A–C). The SVM model of the 2d-fALFF per unit thickness maps performed better in terms of classification accuracy, sensitivity, and specificity when compared with 2d-fALFF (Supplementary Tables S5, S6).

We observed a similar performance of the SVM model of 2d-fALFF and 2d-fALFF per unit thickness of the slow-4 and slow-5 frequency bands in the acute and remitting MS patients and HCs, as shown in Figures 5D–I. Especially in the slow-4 frequency band, compared with 2d-fALFF, the SVM model of 2d-fALFF per unit thickness had significantly higher classification performance in distinguishing between remitting and acute MS using the Delong test (*p* < 0.001) (see Supplementary Table S5).

## 4. Discussion

This study showed that alterations of the 2d-fALFF values and cortical thickness values were detected in some brain regions of MS patients. Moreover, cortical thickness was shown to have some effects on the between-group comparison, clinical correlation, and classification analyses of 2d-fALFF in MS patients. According to the additional SVM classification, 2d-fALFF per unit thickness can be utilized more as a potential marker to distinguish among remitting MS, acute MS, and HCs than 2d-fALFF values. Interestingly, an impact on the effect of cortical thickness on 2d-fALFF values also occurred in the subfrequency band, particularly in the slow-4 band.

### 4.1. Between-group comparison of cortical thickness maps

In this study, it was found that acute MS patients had reduced cortical thickness in some brain regions (L-posterior opercular cortex, L-orbital, and polar frontal cortex), increased cortical thickness in some brain regions (L-fourth visual areal cortex), and remitting MS patients had reduced cortical thickness in some brain regions (L-inferior parietal cortex, R-ventral stream visual cortex). The mechanism of cortical thinning in MS is still unclear. Studies have speculated that the decreased cortical thickness might result from the reductions in dendritic arborization or neuronal cell atrophy in the grey/white matter (35), which needs to be further studied in the future. For the results of some brain regions with increased cortical thickness, we speculate that there may be two reasons. One is that multiple sclerosis is characterized by inflammation and demyelination, with blood–brain barrier disruption, focal leukocyte infiltration, microglial activation, pro-inflammatory cytokine release, mitochondrial oxidative damage, and iron depositions (36). However, studies have shown MS patients with the glial–lymphatic system impaired (37), cerebrospinal fluid and interstitial fluid return blocked, and interstitial clearance reduced, leading to the accumulation of inflammatory and neurotoxic elements, such as meningeal T cells and toxic cytokines (e.g., the chemokine ligand, the interferon-γ, the tumor necrosis factor, and lymphotoxin-α/β), resulting in neuronal swelling. Moreover, there is also the activation of brain areas by repeated stimulation, a phenomenon that has been interpreted as a beneficial functional reorganization for MS to limit clinical impairment.

### 4.2. Between-group comparison of 2d-fALFF and 2d-fALFF per unit thickness maps

In this study, we discovered a decreased signal in the medial temporal cortex in remitting MS compared with acute MS and reduced right paracentral lobular and mid-cingulate cortex and right dorsal stream visual cortex signals in remitting MS compared with HC. In early stages of lesion or the acute phase, axons and neurons are in part preserved; however, with the maturation of the lesions and chronicity of the disease, substantial axonal loss is seen, and the susceptibility of the target tissue for neurodegeneration increases (38). We noticed some similar changes in MS patients’ acute and remitting phases compared with HC, such as increased 2d-fALFF values in the visual-related cortex. We hypothesize that the findings of this study may be due to a compensatory increase in visual information input and a compensatory increase in the processing areas of patients with acute and remitting MS who call on more resources. Visual impairment is a common manifestation of multiple sclerosis with inflammatory, demyelinating, and neurodegenerative lesions that affect afferent and efferent visual function (39, 40). However, some of the visual function areas are very small, likely due to the mild disability of the enrolled MS patients. The dorsolateral prefrontal cortex is the center of the premotor area and cognitive function, as shown in a previous study of fALFF in MS (41), and it has been suggested that “increased connectivity between the dorsolateral prefrontal and sensory cortices may be associated with MS-related fatigue” (42). The increased signal in the dorsolateral prefrontal cortex of remitting MS showed that these brain regions were used to compensate for the potential cognitive impairment of MS to maintain functional stability.

By dividing functional value by the cortical thickness, the influence of cortical thickness on the identification accuracy of 2d-fALFF in MS patients can be reduced, which helps us to understand the changes of neuronal oscillations of unit cortical thickness in MS patients. Compared with 2d-fALFF, we can see the 2d-fALFF increased from no signal to a positive signal in the right inferior frontal cortex for remitting MS vs. acute MS in 2d-fALFF per unit thickness maps. Evidence suggests that the right inferior frontal cortex plays a specialized role in response inhibition and a broader role for this region in attentional control (43). Parisi et al. investigated how resting state functional connectivity of the anterior cingulate cortex (ACC) correlates with cognitive rehabilitation in relapsing remitting MS patients and ACC activity correlates with the inferior frontal gyrus (44). Interesting, we also found that 2d-fALFF increased from a negative signal to no signal in the right paracentral lobule and middle cingulate cortex for remitting MS vs. HC and the right medial temporal cortex in remitting MS vs. acute MS. No significant cortical thickness changes were found in these brain regions, which may be due to the confusion of structural changes, large coefficient of variation, and high degree of dispersion in MS patients. However, we can still find that cortical thickness affects our observation of functional areas of the brain in MS patients.

### 4.3. Clinical relevance of 2d-fALFF and 2d-fALFF per unit thickness maps

The 2d-fALFF from the regions of the left early visual cortex from remitting MS patients showed a significantly positive correlation with disease duration, suggesting that increased neuronal activity is correlated with prolonged disease duration. Usually, the brain lesion of MS initially causes an increase in connectivity, followed by a subsequent decrease before reaching a plateau (45). Unfortunately, we were probably not able to detect this phenomenon as we studied subjects with heterogeneous disease duration, thus including patients at different stages of the disease. Hence, the brain neuronal oscillations alteration present in our study should be interpreted with caution. It may be that patients with longer disease histories show greater compensatory capacity in these areas. Compared with 2d-fALFF, 2d-fALFF per unit thickness of the right ventral stream visual cortex in the typical band was positively correlated with MFIS scale scores in the acute MS group, and the increased left early visual cortical area in the slow-5 band was positively correlated with the EDSS scores and MFIS scores. The bias in the correlation has also been seen in previous studies (46), and the exact cause of this bias is unknown and may be related to complex brain function compensations. It may be suggested that the increased synchronization between brain regions may not necessarily be beneficial to MS and may be a failed attempt to restructure the network in order to overcome structural damage. Furthermore, Sbardella et al. have argued that the clinical correlation with abnormal voxels of medial visual areas is likely MS specific (47).

### 4.4. Classification analysis in 2d-fALFF and 2d-fALFF per unit thickness maps

Surface-based fALFF and surface-based fALFF per unit thickness both performed remarkably well in the SVM analysis for detecting MS patients, especially in the remitting phase. Therefore, surface-based fALFF might be a novel potential imaging biomarker of MS. Despite the fact that volume-based functional metrics are frequently used to examine functional changes in a range of disorders, they ignore the intersubject diversity in cortical folding patterns. However, in the surface-based space, anatomical regions can be precisely matched with identical locations in the reference template, reducing intersubject variability and boosting statistical power (48–50).

Compared with 2d-fALFF, the SVM model of 2d-fALFF per unit thickness could better distinguish acute MS patients. This may be due to the high individual variability of cortical folding patterns and the many relatively wrinkled boundary regions, especially in MS patients with brain atrophy, which, after spatial smoothing, greatly weakens the fidelity of regional assignment and hinders our observation of functional brain regions. Taken together, 2d-fALFF per unit thickness could be a potential imaging biomarker for MS patients.

### 4.5. The effect of cortical thickness on 2d-fALFF values in the subfrequency band

Different oscillation frequencies showed specific peculiarities and were involved in different aspects of brain functions. Gray matter-related oscillations primarily occurred in the slow-4 and slow-5 range. In addition, slow-4 fluctuations were more robust in the basal ganglia than slow-5, while slow-5 was more dominant within ventromedial prefrontal cortices than slow-4. Although robust for both, test–retest reliability was greater and more widely distributed for slow-4 than for slow-5 (25). Several regions exhibited significant differences in 2d-fALFF values in all the subfrequency bands. These results indicate that the alterations in regional activity in MS patients are frequency dependent. In both acute and remitting MS, we continued to detect alterations in visually relevant brain regions in the subfrequency band, pointing to a complicated process of visual function impairment and adaptability in the brains of MS patients. We observed increased 2d-fALFF values in the right dorsolateral prefrontal cortex and right lateral temporal cortex in the slow-5 band in remitting MS. The lateral temporal cortex is closely associated with human cognitive executive activity as part of the default mode network (51). The 2d-fALFF values of the left paracentral lobule and middle cingulate cortex and right anterior cingulate and medial prefrontal cortex were detected in the slow-4 band in remitting MS. The middle cingulate cortex serves as a key region in the multimodal network that integrates information from primary sensory areas (e.g., visual, auditory, and somatosensory) (52). Interestingly, an increased signal of the left auditory contact cortex was observed only in the slow-4 band in remitting MS, although no correlation was found with the PASAT scale scores, still suggesting adaptive functional reorganization in response to reduced auditory attention in patients with MS in the remitting phase. We only observed an increased signal in the inferior parietal cortex in the slow-4 band in patients with acute MS. The inferior parietal cortex plays a specific role in the performance of fatigue and sustained attention tasks (53), suggesting that this change could be specific to acute MS. This finding suggests a frequency-specific alternating characterization of intrinsic brain activity. Impaired motor function is one of the most common clinical symptoms in MS. Our findings suggested damage to motor-related cortical brain areas in acute MS patients.

Compared with 2d-fALFF, acute MS patients also had more increased regions in 2d-fALFF per unit thickness maps both in the slow-4 and slow-5 bands, such as 2d-fALFF, which increased from no signal to positive signals in the left primary visual cortex in the slow-4 band and right inferior parietal cortex in the slow-5 band. We have described previously that acute MS called in a large number of compensatory behaviors of the visual cortex. This discovery might be explained by a phenomenon known as brain compensation, in which extra resources are used by the brain to maintain performance at the same level as before. The widespread white matter and gray matter damage associated with MS is related to the inflammatory demyelination and axonal degeneration in the central nervous system (54). This finding’s greater neuronal effort to counteract the harmful effects on the structure and function of the human brain is another possible explanation. Some brain regions saw an apparent increase in signal, which, once we account for the local thickness, disappear, such as the positive 2d-fALFF signals to no signal in the right lateral temporal cortex, left primary visual cortex, left dorsolateral prefrontal cortex, and right anterior cingulate and medial prefrontal cortex. Therefore, the 2d-fALFF values per unit cortical thickness in these brain regions did not increase. Although no changes in cortical thickness were found in these brain regions, it still suggests that the effect of cortical thickness should be considered when observing changes of neuronal oscillations in MS patients.

In both the slow-4 and slow-5 bands, MS and HC could be distinguished well by 2d-fALFF and 2d-fALFF per unit thickness. Intriguingly, the 2d-fALFF per unit thickness in the slow-4 band could more effectively separate MS patients in the acute phase from those in remitting. This may be due to the following reasons. The slow-5 band, which has more power, is associated with the integration of large-scale neural networks and long-distance connectivity, whereas the slow-4 band, which has less power, is linked with more local neural activity and short connections (25). However, there is currently insufficient evidence that relapses in MS are associated to an increase in neural activity. Therefore, further studies are needed to further elucidate the sensitivity and specificity of slow-4 in MS patients.

There are some limitations in this study. First, this study only explored the effect of cortical thickness on patients with acute and remitting MS based on the cortex, focusing only on the cortex and not on the deep gray matter region; the latter is also a key region in MS that regulates cognitive function. Second, in addition to cortical thickness, cortical lesions are also known to affect connectivity (across all frequency band) (55). Finally, most of the patients included in this study had only mild disability and relatively low EDSS scores, which was due to scanning safety and patient tolerability considerations.

## 5. Conclusion

In the present study, we found 2d-fALFF and cortical thickness alterations in many brain regions of MS patients. The between-group comparison, clinical correlation, and classification analysis showed that 2d-fALFF in MS patients can be potentially influenced by cortical thickness, which should be considered in future works. In addition, 2d-fALFF per unit thickness can be utilized as a potential marker to distinguish among remitting MS, acute MS, and HCs.

## Data availability statement

The datasets presented in this article are not readily available because of ethical and privacy restrictions. Requests to access the datasets should be directed to XL, 2273322450@qq.com.

## Author contributions

XL: conceptualization, data curation, software, writing – original draft, visualization, and writing – review and editing. LeW: conceptualization, resources, investigation, and data curation. YZ, YW, and TH: methodology and data curation. LiW: methodology. MH: data curation. FZ: project administration, supervision, and writing – review and editing. All authors contributed to the article and approved the submitted version.

## Funding

This work was supported by the program for National Natural Science Foundation of China (82160331 and 81771808).

## Conflict of interest

The authors declare that the research was conducted in the absence of any commercial or financial relationships that could be construed as a potential conflict of interest.

## Publisher’s note

All claims expressed in this article are solely those of the authors and do not necessarily represent those of their affiliated organizations, or those of the publisher, the editors and the reviewers. Any product that may be evaluated in this article, or claim that may be made by its manufacturer, is not guaranteed or endorsed by the publisher.

## Footnotes

^1^ http://rfmri.org/DPABISurf

^2^ https://www.csie.ntu.edu.tw/~cjlin/libsvm/
