# Supplementary material for: Altered neural intrinsic oscillations in patients with multiple sclerosis: effects of cortical thickness
Source: Front Neurol. 2023 Sep 25;14:1143646. doi: 10.3389/fneur.2023.1143646 (PMC10560735; doi:10.3389/fneur.2023.1143646)
Supplement: Supplementary file 1 [file Data_Sheet_1.docx]

supplementary materials


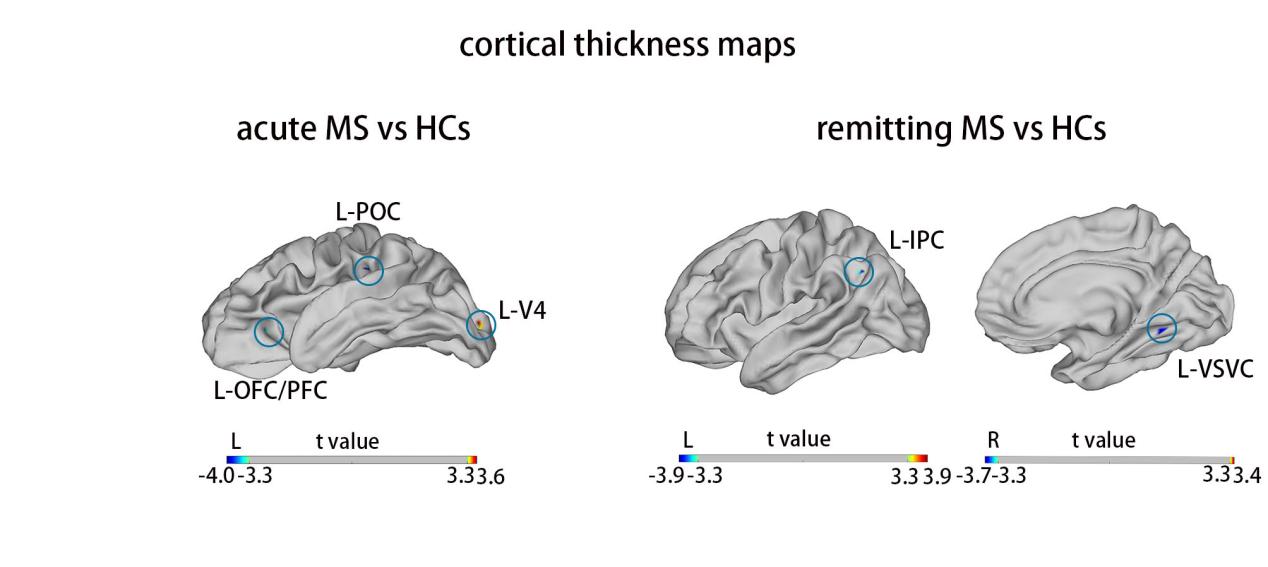


Fig. 1. Brain regions with significant differences in cortical thickness maps among acute MS, remitting MS and HCs.

Note: L=left; R=right; OFC/PFC=orbital and polar frontal cortex; POC=posterior opercular cortex; V4=fourth visual areal cortex; IPC=inferior parietal cortex; VSVC=ventral stream visual cortex; blue circle=brain regions with significant differences in cortical thickness maps.


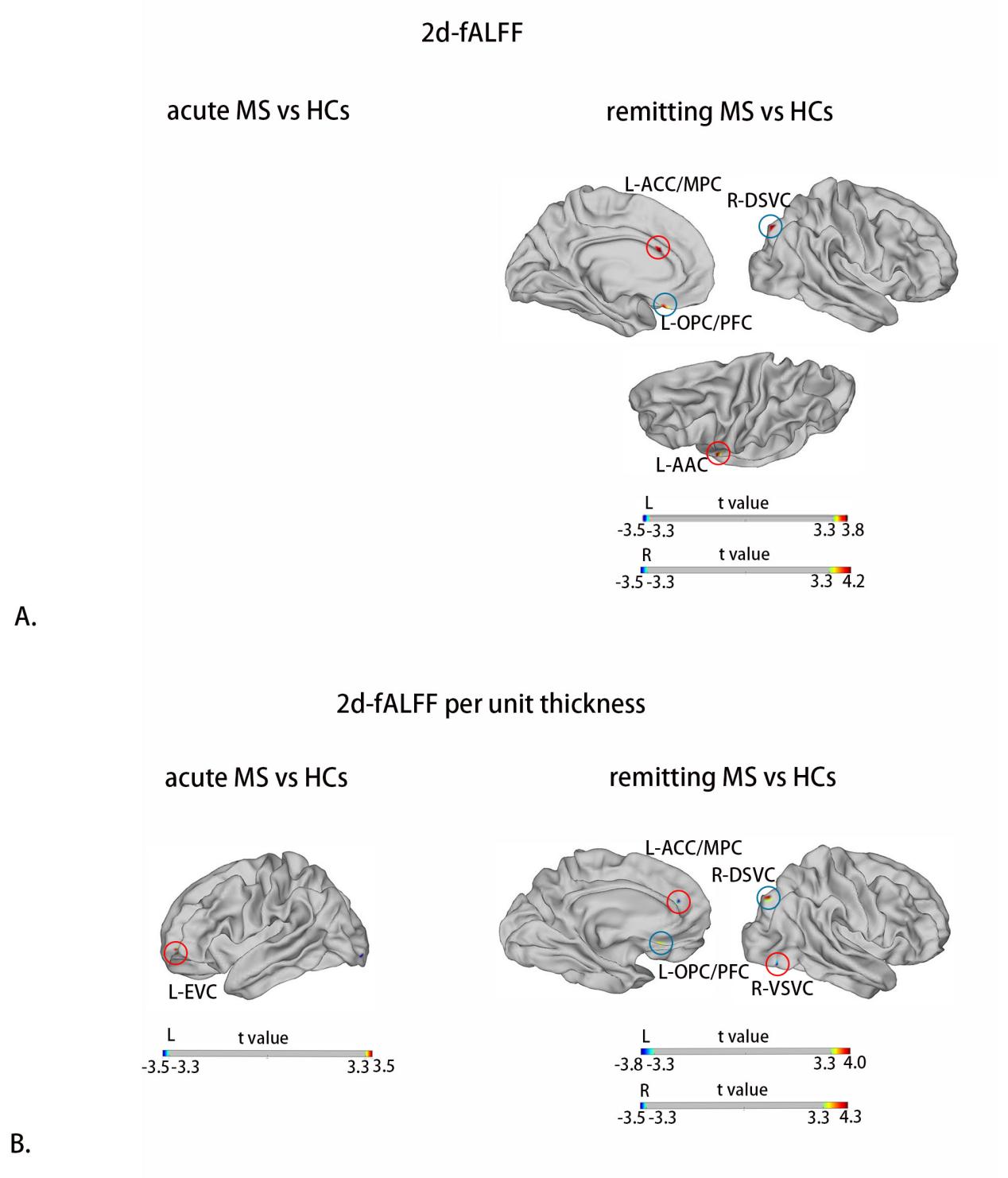


Fig. 2. Brain regions with significant differences in 2d-fALFF maps (A) and 2d-fALFF per unit thickness maps (B) in the slow-2 band among acute MS, remitting MS and HCs.

Note: L=left; R=right; OFC/PFC=orbital and polar frontal cortex; ACC/MPC=anterior cingulate and medial prefrontal cortex; AAC=auditory association cortex; DSVC=dorsal stream visual cortex; VSVC=ventral stream visual cortex; EVC=early visual cortex; red circle=different regions between 2d-fALFF maps and 2d-fALFF per unit thickness maps; blue circle=similar regions between 2d-fALFF maps and 2d-fALFF per unit thickness maps.


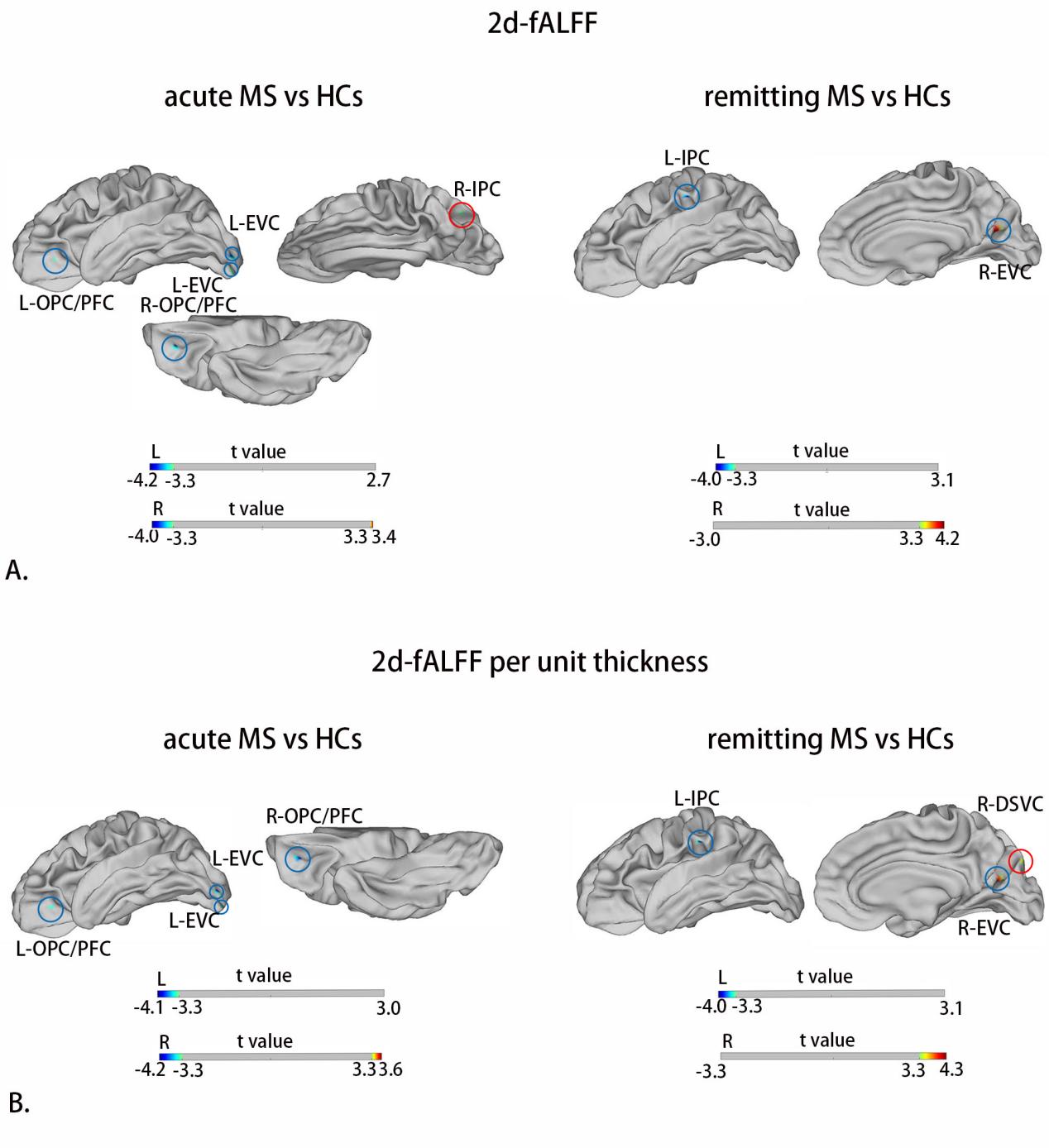


Fig. 3. Brain regions with significant differences in 2d-fALFF maps (A) and 2d-fALFF per unit thickness maps (B) in the slow-3 band among acute MS, remitting MS and HCs.

Note: L=left; R=right; IPC=inferior parietal cortex; EVC=early visual cortex; DSVC=dorsal stream visual cortex; OPC/PFC=orbital and polar frontal cortex; red circle=different regions between 2d-fALFF maps and 2d-fALFF per unit thickness maps; blue circle=similar regions between 2d-fALFF maps and 2d-fALFF per unit thickness maps.


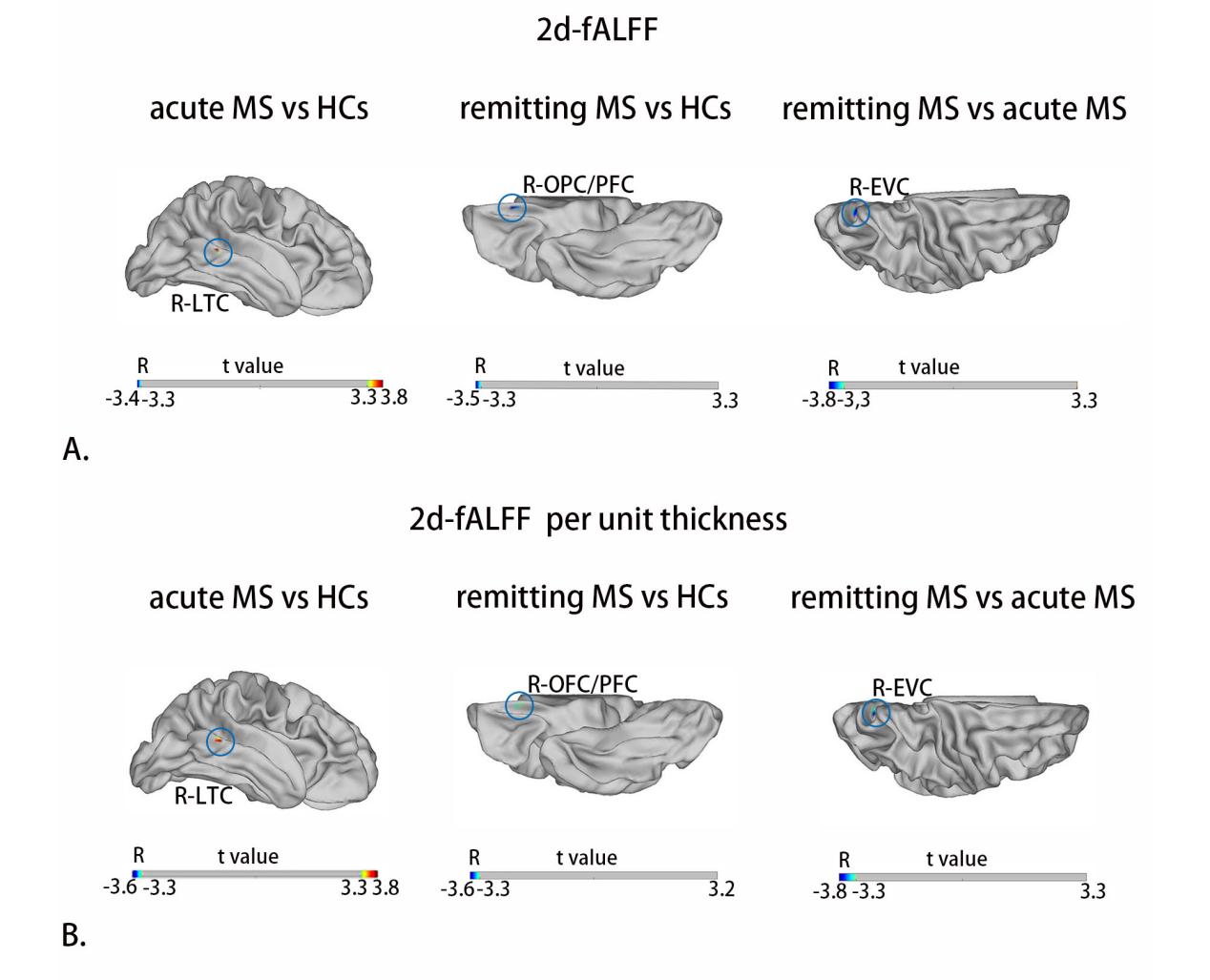


Fig. 4. Brain regions with significant differences in 2d-fALFF maps (A) and 2d-fALFF per unit thickness maps (B) in the slow-6 band among acute MS, remitting MS and HCs.

Note: L=left; R=right; EVC=early visual cortex; OPC/PFC=orbital and polar frontal cortex; LTC=lateral temporal cortex; HCP=Human Connectome Project; MNI=Montreal Neurological Institute; red circle=different regions between 2d-fALFF maps and 2d-fALFF per unit thickness maps; blue circle=similar regions between 2d-fALFF maps and 2d-fALFF per unit thickness maps.

Table 1. Brain regions with significant differences in cortical thickness maps among acute MS, remitting MS and HCs.

|  | Brain regions | HCP | Cluster size (mm^2^) | MNI | | | Peak intensity |
| --- | --- | --- | --- | --- | --- | --- | --- |
|  |  |  |  | X | Y | Z |  |
| acute MS vs. HCs | L-V4 | 6 | 38.578 | -24.58 | -91.59 | 6.48 | 3.57 |
|  | L-POC | 101 | 23.778 | -49.69 | -24.99 | 21.75 | -4.02 |
|  | L-OFC/PFC | 66 | 23.511 | -31.13 | 31.23 | -13.26 | -3.67 |
| remitting MS vs. HCs | L-IPC | 149 | 21.702 | -50.53 | -60.06 | 35.28 | 3.58 |
|  | R-VSVC | 160 | 25.398 | 33.60 | -52.82 | -7.62 | -3.68 |
| acute MS vs. remitting MS | - | - | - | - | - | - | - |

Note: L=left; R=right; OFC/PFC=orbital and polar frontal cortex; POC=posterior opercular cortex; V4=fourth visual areal cortex; IPC=inferior parietal cortex; VSVC=ventral stream visual; blue circle=brain regions with significant differences in cortical thickness maps; HCP=Human Connectome Project; MNI=Montreal Neurological Institute.

Table 2. Brain regions with significant differences in 2d-fALFF maps and 2d-fALFF per unit thickness maps in the slow-2 band among acute MS, remitting MS and HCs.

|  | Brain regions | HCP | Cluster size (mm^2^) | MNI | | | Peak intensity |
| --- | --- | --- | --- | --- | --- | --- | --- |
|  |  |  |  | X | Y | Z |  |
| 2d-fALFF in acute MS vs. HCs | - | - | - | - | - | - | - |
| 2d-fALFF per unit thickness acute MS vs. HCs | L-EVC | 5 | 25.28 | -27.05 | -95.57 | -7.62 | -3.51 |
| 2d-fALFF in remitting MS vs. HCs | L-OFC/MPC | 93 | 28.690 | -3.79 | 25.19 | -25.83 | 3.81 |
|  | L-ACC/MPC | 58 | 28.698 | -6.74 | 21.52 | 22.56 | 3.83 |
|  | L-AAC | 107 | 28.109 | -56.33 | 3.36 | -7.91 | 3.80 |
|  | R-DSVC | 152 | 33.431 | 23.46 | -81.91 | 39.50 | 4.16 |
| 2d-fALFF per unit thickness in remitting MS vs. HCs | L-OFC/PFC | 93 | 24.857 | -3.79 | 25.19 | -25.83 | 3.58 |
|  | L-ACC/PFC | 62 | 19.069 | -13.04 | 39.50 | 21.36 | -3.78 |
|  | R-DSVC | 152 | 46.789 | 23.46 | -81.91 | 39.50 | 4.32 |
|  | R-VSVC | 22 | 22.308 | 44.85 | -78.84 | -10.92 | -3.40 |
| 2d-fALFF in remitting MS vs. acute MS | - | - | - | - | - | - | - |
| 2d-fALFF/thickness in remitting MS vs. acute MS | - | - | - | - | - | - | - |

Note: L=left; R=right; OPC/PFC=orbital and polar frontal cortex; ACC/MPC=anterior cingulate and medial prefrontal cortex; AAC=auditory association cortex; DSVC=dorsal stream visual cortex; VSVC=ventral stream visual cortex; EVC=early visual cortex; HCP=Human Connectome Project; MNI=Montreal Neurological Institute.

Table 3. Brain regions with significant differences in 2d-fALFF maps and 2d-fALFF per unit thickness maps in the slow-3 band among acute MS, remitting MS and HCs.

|  | Brain regions | HCP | Cluster size (mm^2^) | MNI | | | Peak intensity |
| --- | --- | --- | --- | --- | --- | --- | --- |
|  |  |  |  | X | Y | Z |  |
| 2d-fALFF in acute MS vs. HCs | L-EVC | 4 | 73.447 | -30.16 | -93.16 | -11.18 | -4.20 |
|  | L-EVC | 4 | 58.474 | -11.83 | -93.99 | -10.20 | -3.55 |
|  | L-OFC/PFC | 92 | 19.469 | -21.36 | 35.89 | -12.28 | -3.54 |
|  | R-IPC | 145 | 27.854 | 33.73 | -72.14 | 37.25 | -3.47 |
|  | R-OFC/PFC | 91 | 30.304 | 22.04 | 41.36 | -12.33 | -4.09 |
| 2d-fALFF per unit thickness in acute MS vs. HCs | L-EVC | 4 | 73.447 | -30.16 | -93.16 | -11.18 | -4.20 |
|  | L-EVC | 4 | 58.474 | -11.83 | -93.99 | -10.20 | -3.55 |
|  | L-OFC/PFC | 92 | 19.469 | -21.36 | 35.89 | -12.28 | -3.54 |
|  | R-OFC/PFC | 91 | 31.914 | 22.04 | 41.36 | -12.33 | -4.15 |
| 2d-fALFF in remitting MS vs. HCs | L-IPC | 147 | 33.105 | -56.11 | -22.87 | 21.71 | -4.00 |
|  | R-EVC | 4 | 86.042 | 21.14 | -55.74 | 14.33 | 4.19 |
| 2d-fALFF per unit thickness in remitting MS vs. HCs | L-IPC | 147 | 33.105 | -56.11 | -22.87 | 21.71 | -4.00 |
|  | R-EVC | 4 | 86.042 | 21.14 | -55.74 | 14.33 | 4.19 |
|  | R-DSVC | 13 | 22.043 | -13.19 | -85.90 | 37.80 | 3.58 |
| 2d-fALFF in remitting MS vs. acute MS | - | - | - | - | - | - | - |
| 2d-fALFF/thickness in remitting MS vs. acute MS | - | - | - | - | - | - | - |

Note: L=left; R=right; IPC=inferior parietal cortex; EVC=early visual cortex; DSVC=dorsal stream visual cortex; OPC/PFC=orbital and polar frontal cortex; HCP=Human Connectome Project; MNI=Montreal Neurological Institute.

Table 4. Brain regions with significant differences in 2d-fALFF maps and 2d-fALFF per unit thickness maps in the slow-6 band among acute MS, remitting MS and HCs.

|  | Brain regions | HCP | Cluster size (mm^2^) | MNI | | | Peak intensity |
| --- | --- | --- | --- | --- | --- | --- | --- |
|  |  |  |  | X | Y | Z |  |
| 2d-fALFF in acute MS vs. HCs | R-LTC | 133 | 28.413 | 64.34 | -39.51 | -8.44 | 3.77 |
| 2d-fALFF per unit thickness in in acute MS vs. HCs | R-LTC | 133 | 24.271 | 64.34 | -39.51 | -8.44 | 3.82 |
| 2d-fALFF in remitting MS vs. HCs | R-OFC/PFC | 93 | 27.265 | 7.49 | 33.62 | -23.60 | -3.35 |
| 2d-fALFF per unit thickness in in remitting MS vs. HCs | R-OFC/PFC | 93 | 27.265 | 7.49 | 33.62 | -23.60 | -3.45 |
| 2d-fALFF in remitting MS vs. acute MS | R-EVC | 5 | 56.132 | 8.85 | -74.46 | 22.88 | -3.77 |
| 2d-fALFF per unit thickness in remitting MS vs. acute MS | R-EVC | 5 | 56.132 | 12.01 | -75.64 | 21.80 | -3.82 |

Note: L=left; R=right; EVC=early visual cortex; OPC/PFC=orbital and polar frontal cortex; LTC=lateral temporal cortex; HCP=Human Connectome Project; MNI=Montreal Neurological Institute.

Table 5. Area under the curve (AUC), 95% confidence interval, regularization parameter C between subgroups and support vector machine (SVM) model performance differences in different frequency bands between subgroups (P value).

|  |  | acute MS vs. HCs | | | remitting MS vs. HCs | | | remitting MS vs. acute MS | | |
| --- | --- | --- | --- | --- | --- | --- | --- | --- | --- | --- |
|  |  | AUC | 95% Confidence interval | C | AUC | 95% Confidence interval | C | AUC | 95% Confidence interval | C |
| Typical band | 2d-fALFF | 0.53 | 0.40-0.66 | 2^7.4^ | 0.86 | 0.75-0.93 | 2^0.6^ | 0.57 | 0.42-0.72 | 2^-10^ |
|  | 2d-fALFF per unit thickness | 0.65 | 0.52-0.77 | 2^8.8^ | 0.83 | 0.72-0.91 | 2^10^ | 0.69 | 0.54-0.82 | 2^0.6^ |
|  | P value^a^ | 0.34 | | | 0.31 | | | 0.25 | | |
| Slow-4 band | 2d-fALFF | 0.88 | 0.77-0.95 | 2^7.6^ | 0.82 | 0.71-0.90 | 2^3^ | 0.51 | 0.36-0.66 | 2^-10^ |
|  | 2d-fALFF per unit thickness | 0.84 | 0.73-0.92 | 2^7.8^ | 0.83 | 0.72-0.91 | 2^6.8^ | 0.94 | 0.82-0.99 | 2^-10^ |
|  | P value^a^ | 0.38 | | | 0.83 | | | <0.001 | | |
| Slow-5 band | 2d-fALFF | 0.87 | 0.76-0.94 | 2^10^ | 0.77 | 0.66-0.87 | 2^-10^ | 0.65 | 0.50-0.79 | 2^-10^ |
|  | 2d-fALFF per unit thickness | 0.88 | 0.77-0.95 | 2^4.4^ | 0.72 | 0.60-0.82 | 2^2.2^ | 0.60 | 0.45-0.74 | 2^0.6^ |
|  | P value^a^ | 0.63 | | | 0.24 | | | 0.63 | | |

Note: HCs=healthy controls; MS=multiple sclerosis. ^a^=Delong test

Table 6. Classification efficiency of the support vector machine (SVM) model in different frequency bands between subgroups.

|  |  | acute MS vs. HCs | | | remitting MS vs. HCs | | | remitting MS vs. acute MS | | |
| --- | --- | --- | --- | --- | --- | --- | --- | --- | --- | --- |
|  |  | Accuracy | sensitivity | specificity | Accuracy | sensitivity | specificity | Accuracy | sensitivity | specificity |
| Typical band | 2d-fALFF | 0.63 | 1.00 | 0.45 | 0.77 | 0.81 | 0.79 | 0.51 | 0.56 | 0.75 |
|  | 2d-fALFF per unit thickness | 0.66 | 0.85 | 0.50 | 0.78 | 0.85 | 0.76 | 0.57 | 0.52 | 0.90 |
| Slow-4 band | 2d-fALFF | 0.84 | 0.80 | 0.93 | 0.78 | 0.70 | 0.88 | 0.57 | 0.92 | 1.00 |
|  | 2d-fALFF per unit thickness | 0.87 | 0.65 | 0.98 | 0.75 | 0.82 | 0.74 | 0.57 | 0.93 | 0.90 |
| Slow-5 band | 2d-fALFF | 0.74 | 0.95 | 0.75 | 0.75 | 0.59 | 0.88 | 0.57 | 0.65 | 0.55 |
|  | 2d-fALFF per unit thickness | 0.77 | 0.85 | 0.70 | 0.70 | 0.67 | 0.81 | 0.97 | 0.931 | 0.45 |

Note: HCs=healthy controls; MS=multiple sclerosis.
